# Krukenberg Tumor in Association with Ureteral Stenosis Due to Peritoneal Carcinomatosis from Pulmonary Adenocarcinoma: A Case Report

**DOI:** 10.3390/medicina56040187

**Published:** 2020-04-17

**Authors:** Irina Balescu, Nona Bejinariu, Simona Slaniceanu, Mircea Gongu, Brandusa Masoud, Smarandita Lacau, George Tie, Maria Ciocirlan, Nicolae Bacalbasa, Catalin Copaescu

**Affiliations:** 1 Department of Surgery, “Ponderas” Academic Hospital, 021188 Bucharest, Romania; catalin.copaescu@deltapromedical.ro; 2 “Carol Davila” University of Medicine and Pharmacy, 020021 Bucharest, Romania; nicolae_bacalbasa@yahoo.ro; 3 Department of Pathology, “Santomar Oncodiagnostic”, 400664 Cluj Napoca, Romania; nona.bejinariu@gmail.ro (N.B.); simona.slaniceanu@gmail.ro (S.S.); 4 Department of Oncology, “Ponderas” Academic Hospital, 021188 Bucharest, Romania; 5 Department of Radiology, “Ponderas” Academic Hospital, 021188 Bucharest, Romania; brandusa.masoud@gmail.ro (B.M.); smarandita.lacau@gmail.ro (S.L.); 6 Department of Urology, “Ponderas” Academic Hospital, 021188 Bucharest, Romania; 7 Department of Gastroenterology, “Ponderas” Academic Hospital, 021188 Bucharest, Romania; maria.ciocirlan@gmail.ro; 8 Center of Excellence in Translational Medicine, Fundeni Clinical Institute, 022328 Bucharest, Romania; 9 ”Grigore T Popa” University of Medicine and Pharmacy, 700115 Iasi, Romania

**Keywords:** pulmonary adenocarcinoma, Krukenberg tumors, ureteral stenosis

## Abstract

Krukenberg tumors from pulmonary adenocarcinoma represent an extremely rare situation; only a few cases have been reported. The aim of this paper is to report an unusual such case in which almost complete dysphagia and ureteral stenosis occurred. The 62-year-old patient was initially investigated for dysphagia and weight loss. Computed tomography showed the presence of a thoracic mass compressing the esophagus in association with a few suspect pulmonary and peritoneal nodules, one of them invading the right ureter. A biopsy was performed laparoscopically on the peritoneal nodules. The right adnexa presented an atypical aspect; right adnexectomy was also found. The histopathological and immunohistochemical studies confirmed that the primitive origin was pulmonary adenocarcinoma. Although both peritoneal carcinomatosis and ovarian metastases from pulmonary adenocarcinoma represent a very uncommon situation, this pathology should not be excluded, especially in cases presenting suspect pulmonary lesions.

## 1. Introduction

Krukenberg tumors account for less than 2% of all ovarian carcinomas and represent ovarian metastases that usually originate from mucosecretory signet ring cell adenocarcinomas of the gastrointestinal tract. The most commonly encountered sites are the stomach and the colon [1,2,3]. In less common situations, Krukenberg tumors originate from other primaries such as the breast, small intestine, and appendix [3]. For lung cancer ovarian metastases, data are even scarcer, with only a few cases being reported so far [4,5,6]. Most often, these cases are represented by pulmonary adenocarcinomas (in up to 45% of cases), and the exact mechanism of development is not well understood [6]. The aim of this paper is to report the case of a patient diagnosed with Krukenberg tumor and peritoneal carcinomatosis invading the right ureter originating from pulmonary adenocarcinoma, in which the final diagnostic was established after performing a laparoscopic adnexectomy.

## 2. Case Report

**Figure 1:**
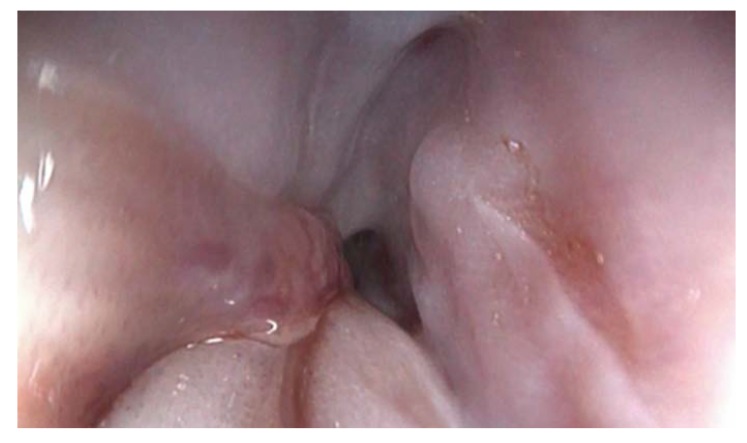
Upper digestive endoscopy revealed an extrinsic compression of the esophagus with normal esophageal lining.

A 62-year-old, nonsmoker woman with no significant pathological antecedents presented to our hospital for almost complete dysphagia. At the time of presentation, the patient was underweight, reporting an approximate weight loss of 15 kg during the last month. During this period, she also observed the apparition of dysphagia first for solids and later also for liquids, which worsened progressively. Biochemical tests demonstrated slow increase of cancer antigen CA 125 levels (74.2 U/mL—units per millilitre), whereas all the other tests (including tumoral markers, urinary and liver tests) were normal. The upper digestive endoscopy raised suspicion of an extrinsic compression of the medial third of the esophagus (at 26 cm from the dental arcade), which did not allow performing the maneuver with a 10 mm endoscope. The stenosis was hardly crossed by using a pediatric 5 mm endoscope, which showed the extension of the affected area on 5 cm. A gastrostomy feeding tube was placed during endoscopy. However, the esophageal lining was normal on the entire surface, again raising suspicion of extrinsic compression (Figure 1).

**Figure 2:**
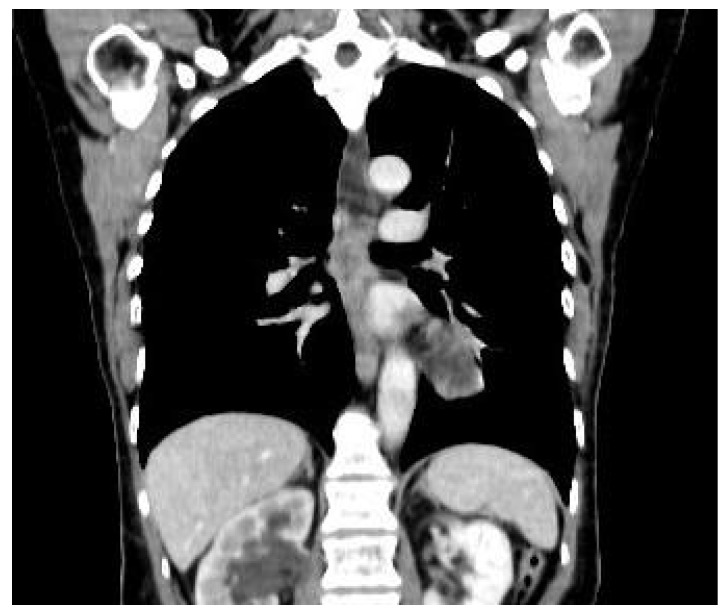
Computed tomography revealing extrinsic compression of the medial third of the esophagus.

**Figure 3:**
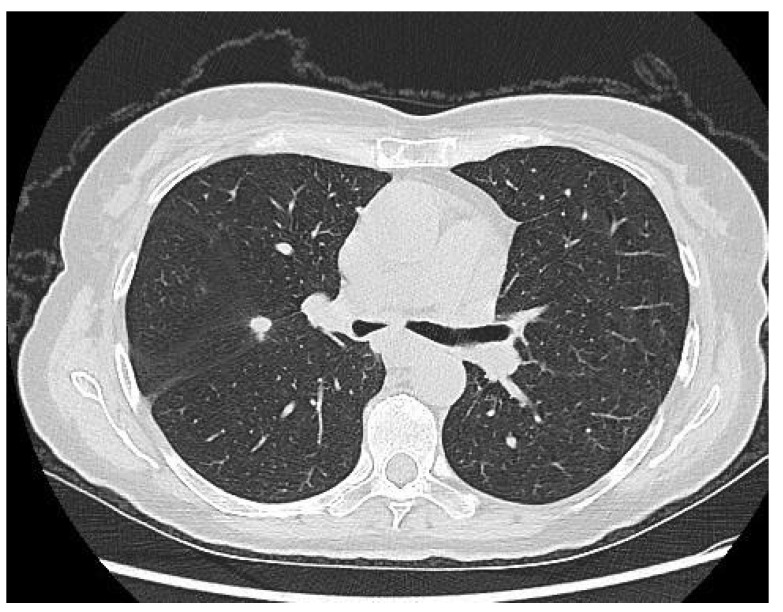
Computed tomography revealing extrinsic compression of the medial third of the esophagus in association with suspect pulmonary nodules.

In this context, an endoscopic ultrasound was attempted to retrieve a biopsy, but the maneuver was unsuccessful due to the extreme compression of the esophagus. The patient later underwent thoracic, abdominal, and pelvic computed tomography that demonstrated the presence of suspect pulmonary nodules in association with a mass compressing the esophagus and invading the pleura, the pericardium, the esophageal wall, and the aortic wall (Figure 2 and Figure 3), as well as a tumoral nodule in close proximity to the uterine cervix invading the right ureter and creating an ureteral stenosis. The cardiologic evaluation demonstrated the presence of a mild pericardial effusion, with no other significant modifications of the cardiac function.

**Figure 4:**
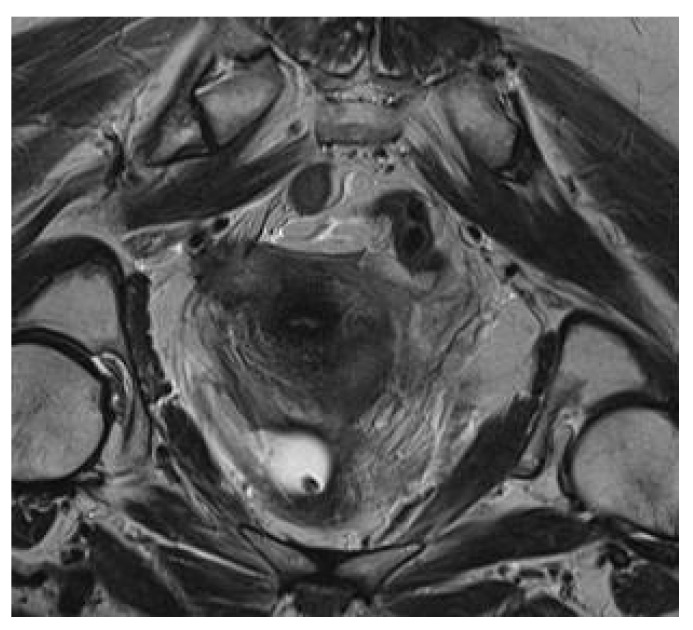
Pelvic magnetic resonance imaging (MRI) revealing the presence of a large tumoral nodule compressing the right ureter.

**Figure 5:**
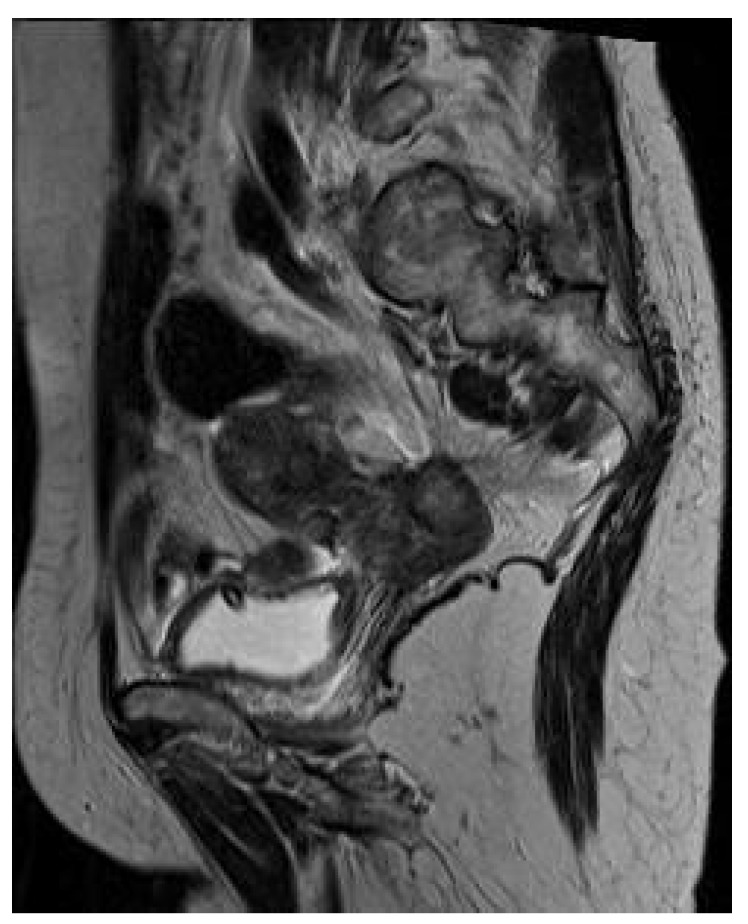
Pelvic MRI revealing the presence of a large tumoral nodule compressing the right ureter.

The imagistic studies were further completed by pelvic magnetic resonance, which raised the suspicion of peritoneal nodules at the pelvic level and confirmed the presence of the tumoral nodule in close proximity to the uterine cervix invading the right ureter and with no apparent connection to the uterine cervix (Figure 4 and Figure 5). In the meantime, other few-millimeter nodules of peritoneal carcinomatosis were found in the pelvic area with no other suspect aspects. The gynecological examination confirmed the presence of a tumoral mass of 3/1.5 cm invading the right ureter, developed in close proximity to the uterine cervix but with no apparent appurtenance to the gynecological tract. No other pathological images were encountered, and the Papanicolaou test failed to demonstrate any modification.

**Figure 6:**
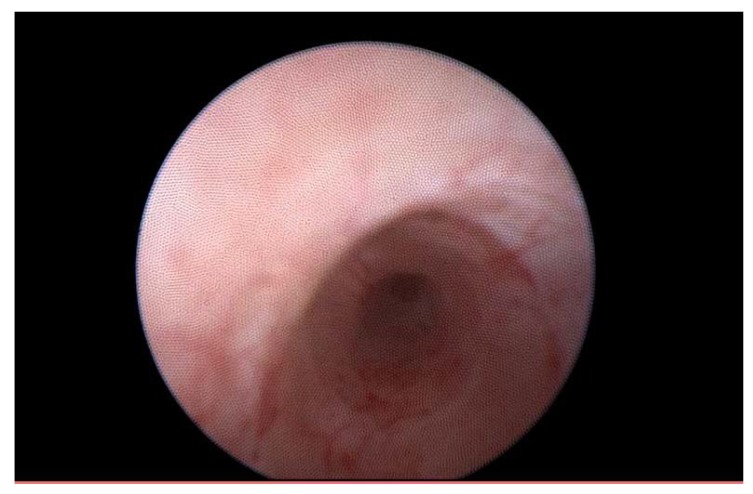
Right ureteroscopy presenting right ureteral stenosis probably due to external compression, a normal aspect of the ureteral lining.

**Figure 7:**
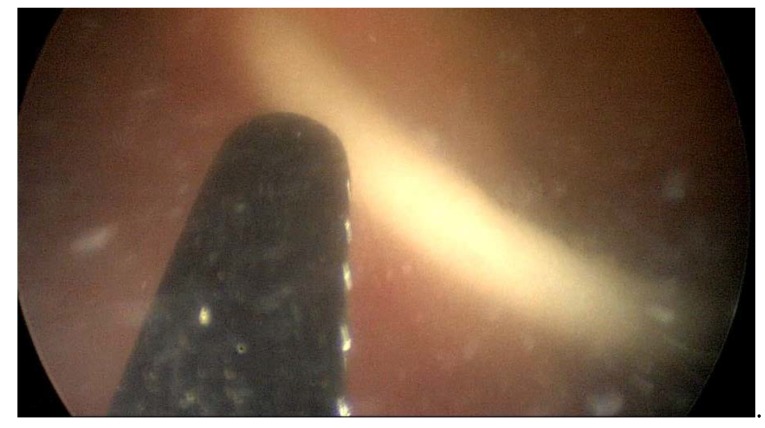
Intravesical positioning of the right Cook catheter.

**Figure 8:**
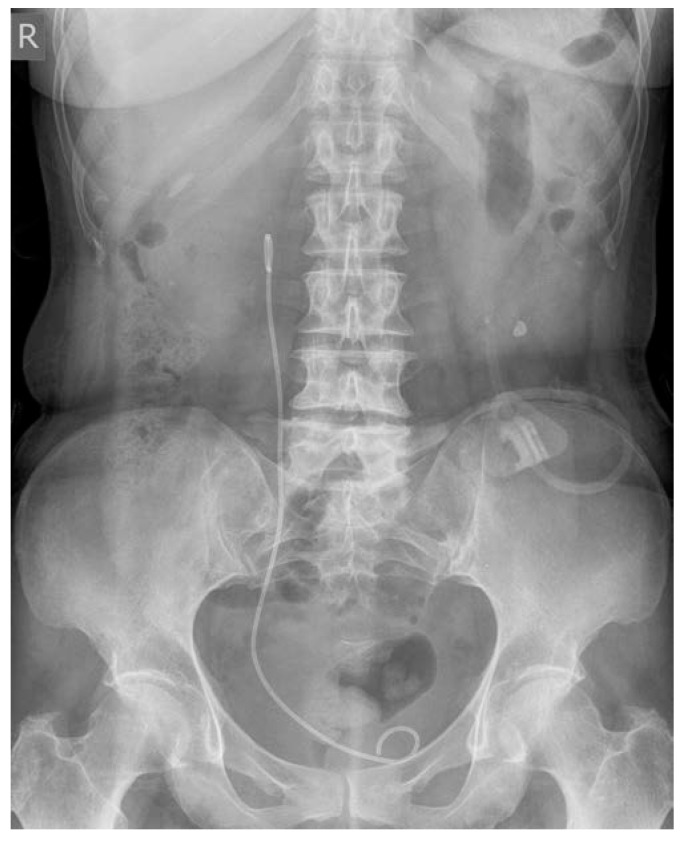
Abdominal X-ray revealing the correct position of the Cook catheter.

Due to an important ureteral stenosis being found, the patient also underwent Cook catheter placement on the right side. At the time of the catheter placement, a cisto-ureteroscopy was performed, which demonstrated an extrinsic compression of the right ureter, with the ureteral and urinary bladder mucosa being normal (Figure 6, Figure 7 and Figure 8). 

**Figure 9:**
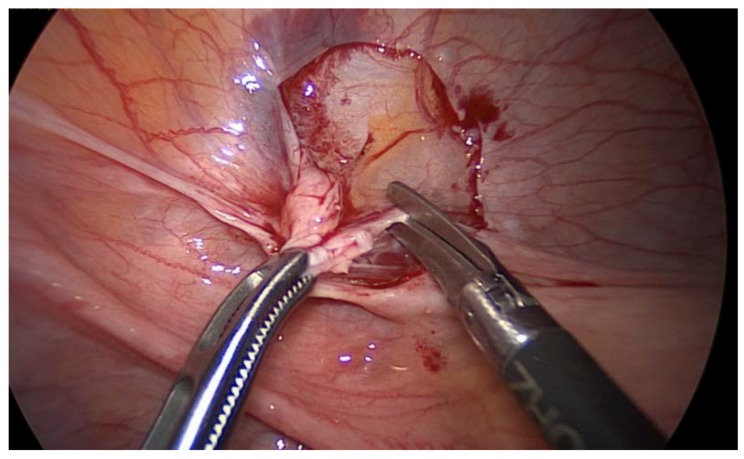
Laparoscopic biopsy of a peritoneal nodule on the anterior surface of the left broad uterine ligament.

**Figure 10:**
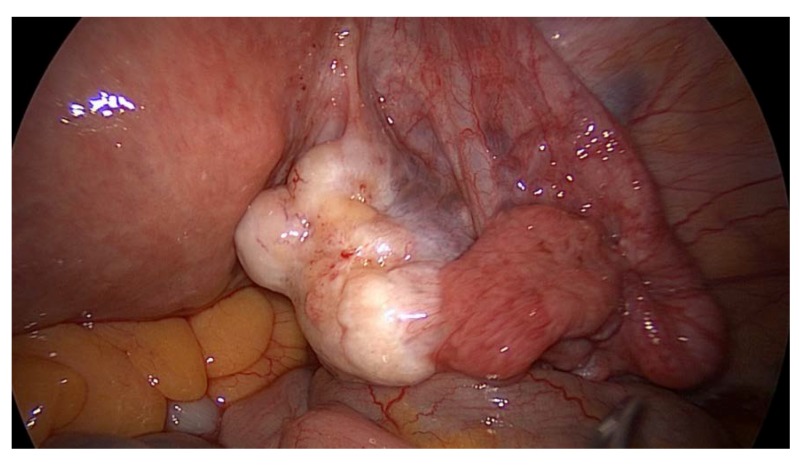
Laparoscopic exploration revealed tumoral transformation of the right adnexa, so a right adnexectomy was performed.

**Figure 11:**
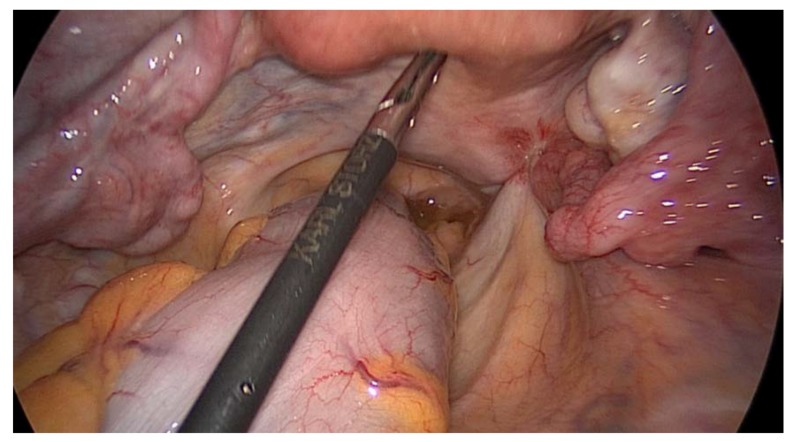
Laparoscopic exploration revealing the nodule of peritoneal carcinomatosis invading the right ureter.

A diagnostic laparoscopy was performed on the patient, which demonstrated the presence of a few peritoneal nodules at the level of the pelvic area, retrieved in association with the nodule invading the right ureter. However, the right ovary was also found to have a pathological aspect, so it was retrieved by performing a right adnexectomy (Figure 9, Figure 10 and Figure 11). 

**Figure 12:**
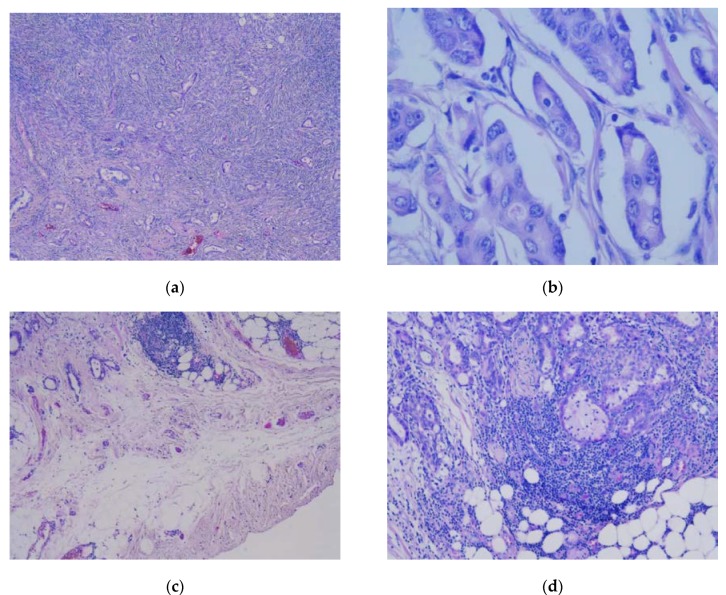
Histopathological study. (**a**) Hematoxylin and eosin (H&E) staining of the ovarian parenchyma revealing tumoral infiltration in more than 90% of the parenchyma (5×); (**b**) H&E staining of the ovarian parenchyma revealing tumoral infiltration in more than 90% of the parenchyma (40×); (**c**) H&E staining of the peritoneum revealing the presence of tumoral deposits (5×); (**d**) H&E staining of the peritoneum revealing perineural invasion (10×).

**Figure 13:**
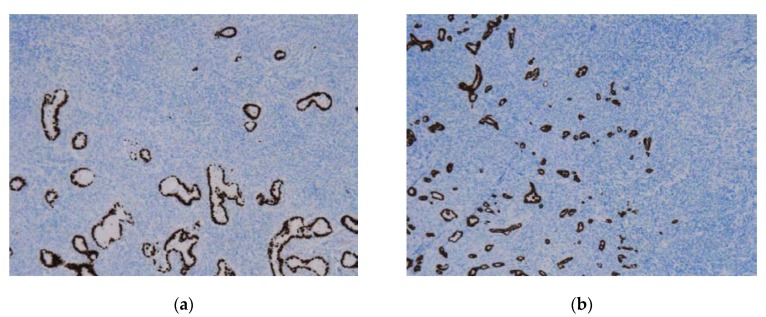

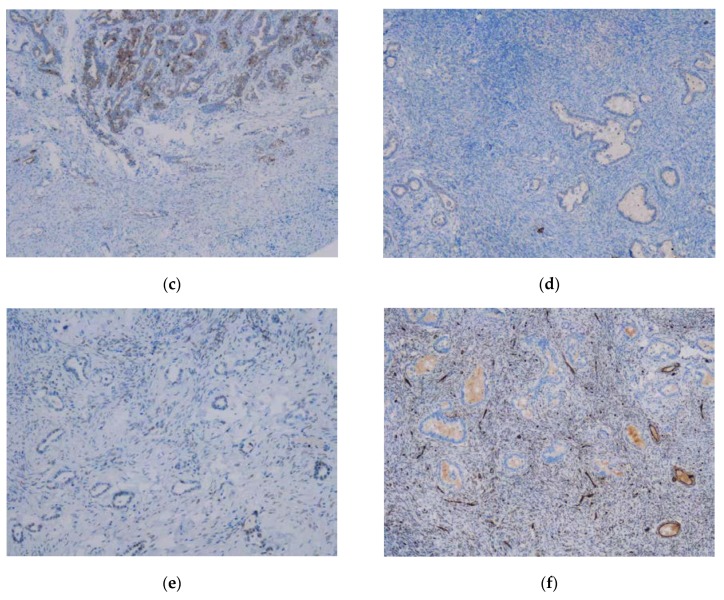
Immunohistochemical study revealing diffuse positivity. (**a**) TTF-1 staining; (**b**) CK7 staining; (**c**) CEA staining; (**d**) PAX8 staining; (**e**) ER staining; (**f**) WT1 staining.

The final histopathological studies of the specimens demonstrated the presence of a well-differentiated adenocarcinoma, with the ovarian parenchyma presenting tumoral invasion on up to 90% of the volume (Figure 12a–d). The immunohistochemical studies established the final diagnostic of pulmonary adenocarcinoma (diffuse and intense positivity for cytokeratin 7 (CK7) and thyroid transcription factor-1 (TTF-1); positivity for carcinoembryonic antigen (CEA); poor positivity for estrogen receptors (ERs); negativity for GATA3 transcription factor, paired-box gene 8 (PAX8), Wilms’ tumor 1 (WT1) factor, homeobox protein (CDX2), p16-cyclin-dependent kinase inhibitor 2A, and S-100 proteins; Figure 13a–f). In this context, the genital, breast, gastric, colonic, esophageal, and pancreatic malignancies were excluded, with the final aspect pulmonary adenocarcinoma. Epidermal growth factor receptor (EGFR), anaplastic lymphoma kinase (ALK), and programmed cell death ligand 1 (PD-L1) tests were performed demonstrating the presence of mutations, so the patient was later confined to the medical oncology clinic for biological treatment. 

## 3. Discussion

Krukenberg tumors were initially described by Friedrich Ernst Krukenberg in 1896, when he revealed the first five such cases [7]. Since then, this histopathological finding has been treated as a separate entity, and further studies demonstrated that the most common origins of the primaries that might lead to the development of such tumors are represented by gastric cancer (up to 76% of cases), followed by colorectal cancer (up to 11% of cases). Other possible origins include the breast, biliary system, pancreas, urinary tract, and gynecological tract [2,3]. Most often, Krukenberg tumors are found bilaterally, and in up to half of cases, they are associated with the presence of ascites [8]. As for Krukenberg tumors with lung cancer origins, it is estimated that less than 4% of all lung cancers lead to the development of ovarian metastases, with up to half of them being represented by small cell carcinomas [4]. To establish that an ovarian tumor has a metastatic origin from a primary lung carcinoma, detection of TTF-1 at the level of the ovarian lesion plays a central role [4]. In our case, the identification of this marker at the level of the specimen of adnexectomy enabled us to suspect the pulmonary origin of the metastatic islets that had been found at the level of the ovarian tissue. The final diagnostic in our case was established based on immunohistochemical studies, which were strongly suggestive for pulmonary adenocarcinoma. The lesions proved to have positive staining for TTF1, CK7, and CEA. In a recent study conducted on a group of 665 specimens of resected pulmonary cancer and 425 resected metastases, Vidarsdottir et al. routinely studied the presence of CK7, CK20, CDX2, CK5, p40, p63, TTF-1, napsin A, GATA3, and PAX8, and demonstrated that primary adenocarcinoma of the lung presented strong positive expression for TTF1 in 90% of cases and for napsin A in 84%, whereas only less than 10% of cases were positive for p63, CDX2, CK20, and GATA3. Additionally, 68% of cases with pulmonary adenocarcinoma presented positive staining for CK7, TTF-1, and napsin A and were negative for the other studied parameters [9]. The authors demonstrated that the presence of TTF1 and CK7 could also be used to differentiate pulmonary adenocarcinoma by squamous cell carcinoma. In the latter histopathological subtype, the positivity of TTF-1 and CK7 was demonstrated in only 3% and 44% of cases, respectively [9]. 

The therapeutic strategies in such cases are mainly decided according to the primary tumor; however, due to the metastatic character of Krukenberg lesions, the presence of ovarian metastases is usually considered as a sign of a systemic disease, which transforms the patient into a candidate for palliative systemic treatment. This pathological finding is usually associated with poor rates of long-term survival [5]. However, in our case, a right adnexectomy was performed as part of the diagnostic strategy and not with curative intent. The patient also showed a large mediastinal mass and peritoneal carcinomatosis nodules invading the ureter as a sign of systemic disease. 

Another particularity of our case is the presence of peritoneal carcinomatosis with a pulmonary adenocarcinoma origin. One nodule of peritoneal carcinomatosis developed in close proximity with the right ureter, where almost complete stenosis had developed. Based on autopsy studies, peritoneal carcinomatosis from pulmonary adenocarcinoma can be encountered in up to 16% of cases; however, clinical manifestation of such lesions are rarely encountered, with obstructive symptomatology being the most common [10,11]. Although this symptomatology is to be expected at the level of the gastrointestinal tract due to the presence of mesenteric nodules, in rare cases, other abdominal structures might be involved. In the case we presented here, obstruction of the right ureter caused a grade III uretero-hydronephrosis. In our case, during laparoscopy, no other nodules of peritoneal carcinomatosis were found, except for few-millimeter lesions at the level of the pelvic area, especially on the uterine serosa and its ligaments.

The rarity of peritoneal carcinomatosis from lung cancer was also demonstrated by the study conducted by Satoh et al., which included 1041 cases with pulmonary cancer over a period of 26 years. Among these cases, only eight patients developed clinically evident peritoneal carcinomatosis [12]; however, regarding histopathological subtypes of pulmonary cancer that present the highest risk of developing peritoneal metastases, adenocarcinoma seems to play the most important role. In another more recent study conducted on the issue of peritoneal carcinomatosis from non-small cell lung carcinoma, Nassereddine et al. included 12 patients diagnosed with peritoneal carcinomatosis from non-small cell lung carcinoma, with 11 cases diagnosed with pulmonary adenocarcinoma. The authors underlined that all cases presented other metastases at the time of the initial diagnosis. The most commonly encountered sites for the metastatic disease were represented by pleura, bone, adrenal glands, liver, and colon. These authors also reported the presence of ovarian metastases, but the incidence of this finding was not reported. In this case series, the most commonly encountered positivity of the immunostaining was the one for PD-L1 (in 37.5% of cases), a finding that was also encountered in the case we reported [13].

The prognosis for these cases seems to be extremely poor, with the overall survival time usually only a few months from the time peritoneal carcinomatosis is diagnosed. Regardless, attention should be paid to identifying the cases that present with EGFR mutation (consisting of exon 19 deletion), which seem to be associated with a more favorable outcome [14]. In such cases tyrosine kinase inhibitors might be administrated, and a significant benefit of survival (of more than 1 year) is expected [15,16]. In the aforementioned case, the presence of EGFR mutation allowed us to introduce this biological treatment with good chances of prolonging the patient’s survival. In addition, a differential diagnostic of the primary tumor with pleural mesothelioma should be ruled out; immunohistochemical studies are the cornerstone process by which to establish this differential diagnosis [17]. 

## 4. Conclusions

Association between peritoneal carcinomatosis creating urinary tract obstruction and Krukenberg tumor is an extremely rare eventuality in patients with pulmonary adenocarcinoma. The case we reported presents a series of particularities: the symptomatology at the time of presentation, almost complete dysphagia due to a thoracic mass compressing the esophagus, the modality in which the final diagnostic was established, and laparoscopic adnexectomy in association with peritoneal nodules biopsy, which revealed positive immunohistochemical staining for TTF-1, CK7, and CEA. The demonstration of EGFR, PD-L1, and ALK mutations offered a chance for improved survival for our patient, who thereby became a candidate for immunotherapy.
